# Extrasynaptic δ‐GABA_A_ receptors are high‐affinity muscimol receptors

**DOI:** 10.1111/jnc.14646

**Published:** 2019-03-06

**Authors:** Ali Y. Benkherouf, Kaisa‐Riitta Taina, Pratap Meera, Asko J. Aalto, Xiang‐Guo Li, Sanna L. Soini, Martin Wallner, Mikko Uusi‐Oukari

**Affiliations:** ^1^ Centre of Integrative Physiology and Pharmacology Institute of Biomedicine University of Turku Turku Finland; ^2^ Department of Neurobiology University of California Los Angeles California USA; ^3^ Turku PET Centre Abo Akademi University Turku Finland; ^4^ Turku PET Centre Turku University Hospital Turku Finland; ^5^ Department of Molecular and Medical Pharmacology University of California Los Angeles California USA

**Keywords:** affinity, association, binding, dissociation, GABA_A_ receptors, muscimol

## Abstract

Muscimol, the major psychoactive ingredient in the mushroom *Amanita muscaria*, has been regarded as a universal non‐selective GABA‐site agonist. Deletion of the GABA_A_ receptor (GABA_A_R) δ subunit in mice (δKO) leads to a drastic reduction in high‐affinity muscimol binding in brain sections and to a lower behavioral sensitivity to muscimol than their wild type counterparts. Here, we use forebrain and cerebellar brain homogenates from WT and δKO mice to show that deletion of the δ subunit leads to a > 50% loss of high‐affinity 5 nM [^3^H]muscimol‐binding sites despite the relatively low abundance of δ‐containing GABA_A_Rs (δ‐GABA_A_R) in the brain. By subtracting residual high‐affinity binding in δKO mice and measuring the slow association and dissociation rates we show that native δ‐GABA_A_Rs in WT mice exhibit high‐affinity [^3^H]muscimol‐binding sites (K_D_ ~1.6 nM on α4βδ receptors in the forebrain and ~1 nM on α6βδ receptors in the cerebellum at 22°C). Co‐expression of the δ subunit with α6 and β2 or β3 in recombinant (HEK 293) expression leads to the appearance of a slowly dissociating [^3^H]muscimol component. In addition, we compared muscimol currents in recombinant α4β3δ and α4β3 receptors and show that δ subunit co‐expression leads to highly muscimol‐sensitive currents with an estimated EC_50_ of around 1–2 nM and slow deactivation kinetics. These data indicate that δ subunit incorporation leads to a dramatic increase in GABA_A_R muscimol sensitivity. We conclude that biochemical and behavioral low‐dose muscimol selectivity for δ‐subunit‐containing receptors is a result of low nanomolar‐binding affinity on δ‐GABA_A_Rs.

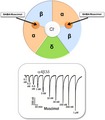

## Graphical abstract

Muscimol has been regarded as a universal non‐selective GABA‐site agonist. Here, we show that δ subunit incorporation leads to a dramatic increase in GABA_A_ receptor (GABA_A_R) muscimol sensitivity. The biochemical and behavioral low‐dose muscimol selectivity for δ subunit‐containing receptors was because of low nanomolar‐binding affinity on α4/6βδ GABA_A_Rs. This paints a consistent picture in which extrasynaptic δ‐GABA_A_Rs are not only exquisitely sensitive to GABA, but also the GABA analogs gaboxadol and muscimol.

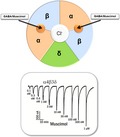

**Abbreviations used**
anova: analysis of variance
BBB: blood–brain barrier
CRC: concentration‐response curve
EC_50_: effective concentration for half maximal activation
EDTA: ethylenediaminetetraacetic acid
GABA_A_R: GABA‐type A receptor
GABA: gamma‐aminobutyric acid
HEK293 cells: human embryonic kidney 293 cells
*K*_D_: equilibrium dissociation constant
*k*_off_: dissociation rate constant
*k*_on_: association rate constant
RRID: research resource identifier
THIP: 4,5,6,7‐tetrahydroisoxazolo[5,4‐c]pyridin‐3‐ol
Tris: tris(hydroxymethyl)aminomethane
WT mouse: wild‐type mouse line
γ2‐GABA_A_R: αβγ2‐type GABA_A_R
δ‐GABA_A_R: αβδ‐type GABA_A_R
δKO mouse: GABA_A_R δ subunit knock‐out mouse line

Gamma‐aminobutyric acid (GABA) is the major inhibitory neurotransmitter in vertebrate brain. The inhibitory action of GABA is mediated via ionotropic GABA_A_ and metabotropic GABA_B_ receptors (Simeone *et al*. 2003). GABA_A_ receptors (GABA_A_R) are pentameric complexes of membrane spanning subunits and belong to the cysteine loop superfamily of ligand‐gated ion channels. GABA_A_R subunits are coded by 19 separate genes, α1‐α6, β1‐β3, γ1‐γ3, δ, ε, π, θ, and ρ1‐ρ3 (Olsen and Sieghart 2008). Most of the GABA_A_R complexes formed in the brain are of type αβγ2 (γ2‐GABA_A_R) with a likely subunit stoichiometry of 2(α):2(β):1(γ2) (Tretter *et al*. 1997; Farrar *et al*. 1999). However, γ2‐GABA_A_Rs, especially those containing α1‐α3 subunits, are clustered at post‐synaptic sites where they mediate fast synaptic phasic inhibition and most of them are sensitive to modulation by benzodiazepines (Olsen and Sieghart 2008). Combinations where δ replaces γ2 (αβδ, δ‐GABA_A_R) reside in extra‐ and perisynaptic membranes where their high GABA sensitivity allows them to be activated by ambient [GABA] to mediate tonic inhibition of the nerve cell (Nusser *et al*. 1998; Brickley *et al*. 1999; Nusser and Mody 2002; Semyanov *et al*. 2004). Here, δ‐GABA_A_Rs are mainly localized in cerebellar granule cells, thalamus (α4β2δ), cerebral cortex (α4β2/3δ), hippocampal dentate gyrus granule cells (α4β2/3δ), caudate‐putamen and in the nucleus accumbens (α4β3δ) (Jechlinger *et al*. 1998; Pirker *et al*. 2000; Pöltl *et al*. 2003).

The functional and pharmacological characteristics of extrasynaptic δ‐GABA_A_Rs are quite different from classical γ2‐GABA_A_Rs. δ‐GABA_A_Rs have much higher affinity for GABA, are insensitive to classical benzodiazepines, show high sensitivity to neurosteroids and Zn^2+^ (Semyanov *et al*. 2004; Mortensen and Smart 2006; Stórustovu and Ebert 2006) and δ‐GABA_A_R‐mediated tonic currents show high sensitivity to ethanol (Hanchar *et al*. 2005; Fleming *et al*. 2007). Recombinantly expressed δ‐GABA_A_Rs show increased maximal currents with the GABA‐analogs 4,5,6,7‐tetrahydroisoxazolo[5,4‐c]pyridin‐3‐ol (THIP, also known as gaboxadol) when compared to GABA. This is likely because of GABA being a partial agonist on these receptors (Bianchi and Macdonald 2003) and δ subunit incorporation dramatically increases their THIP sensitivity (Meera *et al*. 2011). This is consistent with the finding that GABA_A_R δ subunit knock‐out (δKO) mice lose low dose THIP effects on tonic currents in neurons in brain slices and behavioral sensitivity to low doses of THIP (Boehm *et al*. 2006; Chandra *et al*. 2006). Similar to THIP, low‐dose muscimol behavioral effects are also dependent on the presence of α6, α4 and δ subunits, with both α4KO and δKO mice much less sensitive to behavioral muscimol effects, whereas ectopic over‐expression of α6 in mice resulted in increased behavioral muscimol sensitivity (Chandra *et al*. 2010). This clearly indicates that α4/6βδ‐GABA_A_Rs are critical for low‐dose behavioral effects of the GABA analogs THIP and muscimol.

Muscimol, the principal psychoactive constituent of *Amanita muscaria* and related species of mushroom (Krogsgaard‐Larsen *et al*. 1981), is produced from ibotenic acid by decarboxylation (Fig. 1a) and has been considered as a general GABA_A_ agonist that activates all GABA_A_R subtypes (Krogsgaard‐Larsen *et al*. 1979; DeFeudis 1980), including specialized rho‐GABA receptors (Ogurusu *et al*. 1999). However, muscimol shows GABA_A_R selectivity with exceptionally high affinity to δ‐GABA_A_Rs (Quirk *et al*. 1995). In addition, when measured with autoradiography, δKO mice lose high‐affinity (6 nM) [^3^H]muscimol binding in the forebrain sections, with drastically reduced binding in the cerebellum (Mihalek *et al*. 1999, Fig. 1b), indicating that under these experimental conditions muscimol shows much higher affinity for δ‐GABA_A_Rs when compared to abundant γ‐GABA_A_Rs.

**Figure 1:**
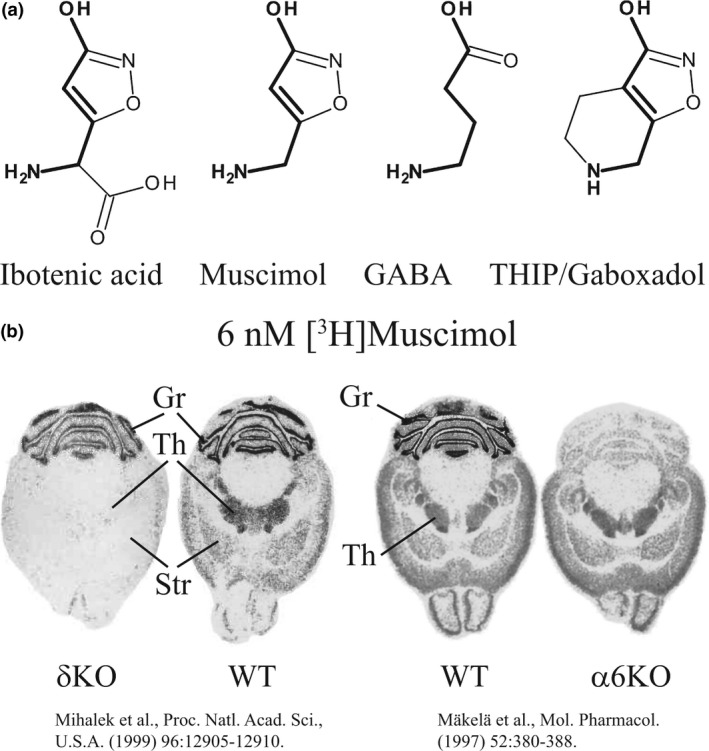
(a) Structures of the muscimol precursor ibotenic acid, GABA and the GABA_A_R agonists muscimol and THIP. The backbone of GABA is shown in bold to illustrate that muscimol and THIP are conformationally restricted GABA analogs. (b) [^3^H]Muscimol (6 nM) autoradiography in brain sections comparing wild‐type (WT) with α6 knockout (α6KO) and delta knockout (δKO) mouse lines. This shows that high‐affinity muscimol binding in the forebrain is δ subunit dependent, wheras in the cerebellum it is α6 subunit dependent. Figure for δKO and the corresponding WT mice are reproduced with permission of the Proceedings of the National Academy of Sciences, U.S.A. (Mihalek *et al*. 1999) and that of α6KO and WT mice with permission of the American Society for Pharmacology and Experimental Therapeutics (Mäkelä *et al*. 1997).

In this study, we investigated high‐affinity (5 nM) [^3^H]muscimol binding in wild‐type (WT) and δKO mouse brains and to several αβγ and αβδ‐type recombinant GABA_A_Rs by measuring binding and unbinding kinetics. Subtraction of residual high‐affinity (5 nM) [^3^H]muscimol binding that is seen on abundant GABA_A_R subtypes in δKO mice from binding in WT membranes allowed us to isolate a native δ‐GABA_A_R component. This isolated component showed very slow muscimol dissociation rate with an apparent *K*_D_ (calculated from *k*_on_ and *k*_off_ rates) for muscimol of 1.6 nM for α4βδ receptors in the forebrain and around 1 nM for α6βδ receptors in the cerebellum. Recombinant α4β3δ receptors expressed in oocytes revealed a biphasic response to muscimol with the high‐muscimol affinity (slowly deactivating/dissociating) component showing an approximate EC_50_ of around 1–2 nM.

We conclude that muscimol is a high‐affinity ligand for both native and recombinant δ‐GABA_A_Rs, providing the molecular basis for the biochemical and behavioral selectivity of muscimol actions on α4/6βδ GABA_A_Rs (Chandra *et al*. 2010).

## Materials and methods

### Animals

Wild‐type (C57BL/6J, WT; RRID: IMSR JAX:000664), and GABA_A_R δ subunit knockout (C57BL/6J, δKO; RRID: MGI:3639693) mice (age 3–12 months, both sexes, University of California at Los Angeles) were used for the studies. However, δKO mice were originally generated by the Homanics lab (Mihalek *et al*. 1999), using ES cell injection into C57BL/6J blastocysts and backcrossed for at least 10 generations with C57BL/6J mice (Jackson Laboratories, stock No. 000664). The mice weighed 19–32 g and they were housed in 12 : 12 h light:dark cycle in static plastic cages in groups of 2–4 mice having *ad libitum* access to Rodent Lab Chow #5001 food and filtered tap water. The animals were killed by decapitation, their brains were removed, the cerebellum was separated with a scalpel from the rest of the brain (i.e., forebrain and midbrain, but loosely referred to here as forebrain), frozen on dry ice and stored at −70°C.

All procedures were in accordance with protocols approved by the University of California at Los Angeles (UCLA) Chancellor's Animal Research Committee (Animal Welfare Approval number: A3196‐01).

### Reagents

[Methylene‐^3^H]muscimol (22 Ci/mmol) was purchased from PerkinElmer Life and Analytical Sciences (Boston, MA, USA, Cat. No. NET 574). Unlabeled muscimol was from Sigma‐Aldrich (St. Louis, MO, USA, Cat. No. M5123). GABA was from Sigma‐Aldrich (Cat. No. A2129).

### Preparation of brain membranes

WT and δKO forebrain and cerebellar membranes were prepared using a modification of the method of Squires and Saederup (2000) essentially as described by Uusi‐Oukari *et al*. (2014). Rat forebrain along with midbrain region was homogenized into 10 mM Tris‐HCl, pH 8.0 buffer containing 2 mM EDTA, using an Ultra‐Turrax T25 (Janke & Kunkel IKA labortechnik) for 20 s at 9500 rpm. The homogenates were centrifuged at 20 000 *g* for 10 min at +4°C and the resulting pellets were washed three times by resuspension and re‐centrifugation with 10 mM Tris‐HCl, pH 8.5 buffer containing 0.2 M NaCl, and 5 mM EDTA. The resulting pellets were then suspended in ice‐cold H_2_O and centrifuged. The pellets were again washed three times with Tris, pH 8.5/NaCl/EDTA as described above. The resulting pellets were finally suspended in assay buffer consisting of 50 mM Tris‐base, pH adjusted to 7.4 with citric acid, and frozen at −70°C. Before a binding experiment, the suspension was thawed, centrifuged, and suspended in assay buffer.

### Recombinant GABA_A_ receptor expression in HEK293 cells and *Xenopus* oocytes

Human embryonic kidney (HEK) 293 cells (ATCC Cat# CRL‐1573; RRID: CVCL_0045; a commonly misidentified cell line by ICLAC; last authenticated by STR DNA profiling in December 2018) were transfected with rat cDNAs (α1, L08490; α6, L08495; β2, X15467; β3, X15468; γ2S, L08497; δ, L08496) in pRK5 plasmids under the control of the cytomegalovirus promoter (Uusi‐Oukari *et al*. 2000), using the calcium phosphate precipitation method essentially as described (Lüddens and Korpi 1997). The plasmids were used in 1 : 1 and 1 : 1 : 1 ratios for transfections containing 2 [(α1 or α6) + (β2 or β3)] or 3 [(α1 or α6) + (β2 or β3) + (γ2S or δ)] different subunits, respectively (5 μg of each plasmid DNA for a 10 cm plate). Mock transfection was done using 5 μg pRK5 plasmid backbone. The cells were harvested 48 h after transfection. Culture medium was removed and the cells were detached from the plates by pipetting in ice‐cold assay buffer containing 2 mM EDTA. The cells were homogenized (Ultra Turrax, 20 s at 9500 rpm), the homogenates centrifuged at 20 000 *g* for 10 min at +4°C, and washed once with the same buffer. The homogenates were finally suspended in assay buffer (1 mL/plate) and either used directly to binding assays or stored frozen at −70°C.

Human α4, α6, β3, and δ cDNA clones for oocyte expression were made by PCR amplification of the coding region (NcoI site introduced with the amplifying 5′ oligonucleotide at the ATG initiation codon) and a HindIII (or SpeI) containing oligo) and cloning it into a NcoI‐HindIII (or SpeI) cut vector backbone derived from pEGFP‐N1 (Addgene 6085‐1). The entire transcribed region was confirmed by sequencing to ensure that protein sequences conform to consensus sequences found in the RefSeq database (https://www.ncbi.nlm.gov/RefSeq). Plasmids were linearized with NotI (New England Biolabs) and cRNA was transcribed, using T7 RNA polymerase (Ambion, mMESSAGE mMACHINE T7 Transcription Kit, Ambion Austin TX, USA, Cat. No. 1344). *Xenopus laevis* (Nasco, product number LM00531) oocytes were prepared from oocyte lobes shared by Dr. Olcese (UCLA, Anesthesiology). The oocytes were injected with 2 ng of each α6 and β3 subunit cRNA alone or together with 10 ng δ cRNA. Currents were measured 5–10 days after injection by two electrode voltage clamp using an Axoclamp 2B amplifier and pCLAMP software. Drug solutions were applied in ND96 (in mM 96 NaCl, 2 KCl, 1.8 CaCl_2_, 1 MgCl_2_, 5 HEPES pH 7.4) by gravity perfusion with bath exchange time of about 2 s. Muscimol was prepared as an aqueous 100 mM stock solution**.**

### Measurement of [^3^H]muscimol binding kinetics

The binding of [^3^H]muscimol (5 nM) was measured in assay buffer at 22°C in a total volume of 300 μL. Triplicate technical replicates of mouse forebrain (190–215 μg protein), cerebellar (180–210 μg protein) or HEK cell (92–132 μg protein) membranes for each time point were incubated with shaking for various times (15 s–15 min) to measure association of the binding. Non‐specific binding was determined in the presence of 100 μM GABA. The incubation was terminated by filtration of the samples with a Brandel Cell Harvester (model M‐24, Gaithersburg, MD, USA) onto Whatman GF/B filters (Whatman International Ltd., Maidstone, UK). The samples were rinsed twice with 4–5 mL of ice‐cold assay buffer. Filtration and rinsing steps took a total time of ~15 s. Air‐dried filters were immersed in 3 mL of Optiphase HiSafe 3 scintillation fluid (Wallac, Turku, Finland) and radioactivity determined in a Wallac model 1410 liquid scintillation counter (Wallac, Turku, Finland). The maximal binding disintegrations per minute (DPM) values (at 15 min in association) for recombinant studies with 5 nM [^3^H]muscimol were between 700 and 2500 DPMs of specific binding (background subtracted). In native membranes, the maximal DPM values were between 2500 and 3000 for WTs and 1300–1500 for δKOs. Mock transfection with pRK5 plasmid did not produce any specific binding over the background.

To measure dissociation of [^3^H]muscimol binding, triplicate technical replicates of each sample of mouse brain or HEK cell membranes for each time point were first pre‐incubated at 22°C in a total volume of 300 μL for 15 min with 5 nM [^3^H]muscimol in the absence and presence of 100 μM GABA. The dissociation was then started by adding 100 μL of 400 μM or 100 μM (non‐specific binding) cold GABA to the incubation mixtures to reach a final 100 μM GABA concentration in all tubes. The tubes were mixed and incubations at 22°C were terminated at various time points (30 s – 30 min) as described above. Dissociation of [^3^H]muscimol from recombinant receptors in HEK cell membranes was also measured at 0–4°C (on ice) to evaluate how fast [^3^H]muscimol dissociates from receptors while washing the filter with ice‐cold assay buffer during filtration.

Saturation analysis of [^3^H]muscimol to WT and δKO mouse forebrain and cerebellar membranes was performed essentially as described by Uusi‐Oukari and Korpi (1989). Triplicate samples of the membranes were incubated in assay buffer with concentration series of hot [^3^H]muscimol (0.1–30 nM) at 0 to 4°C for 30 min in the absence and presence of 100 μM GABA determining the non‐specific binding. The incubations were terminated as described above.

The hypothetical values for binding of [^3^H]muscimol to δ‐GABA_A_Rs in WT animals, ‘*native* δ*‐GABA*_*A*_ *Rs’*, were calculated by subtracting the specific δKO binding values (binding to γ2‐GABA_A_Rs) from the corresponding WT values at each time point: *native* δ*‐GABA*_*A*_ *Rs* = WT‐ δKO. Because of the lack of low‐affinity binding and the relatively small number of time points in our assays, the binding curves fitted better in ‘one binding site’ model. However, varying fast and slow dissociation components are obvious in the graphs (see Figs 3 and 4).

**Figure 2:**
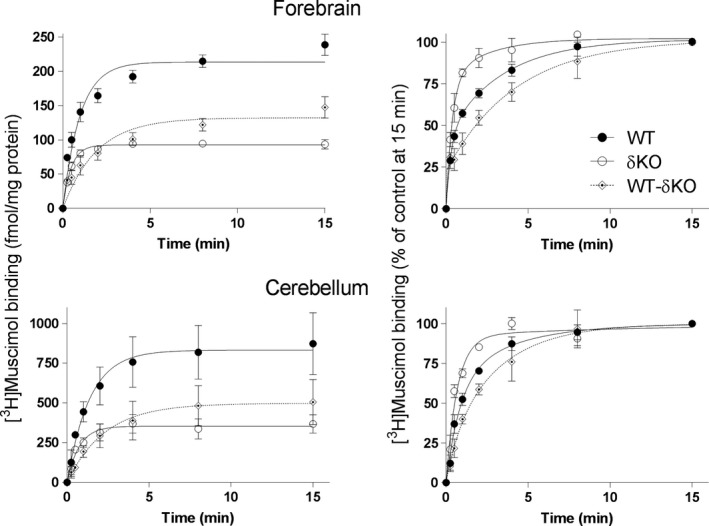
Majority of high‐affinity muscimol binding is δ subunit‐dependent. Association of [^3^H]muscimol binding to forebrain (*n* = 8 independent experiments in both mouse lines using individual forebrains in each experiment) and cerebellar (*n* = 3 independent experiments using pools of 3 individual cerebella from the mouse line in each pool) membranes of WT and δKO mice (mean ± SEM). The experiments were performed in triplicate technical replicates. Forebrain and cerebellar membranes were incubated with 5 nM [^3^H]muscimol alone and in the presence of 100 μM GABA to determine non‐specific binding. The incubations were terminated at various time points by filtration onto GF/B filters. The values are expressed as fmol/mg protein (left panels) and as % of binding at 15 min (right panels). Binding to δ‐GABA_A_Rs (WT‐δKO) was calculated by subtracting binding to non‐δ‐GABA_A_RS in δKO mice from binding to WT membranes.

**Figure 3:**
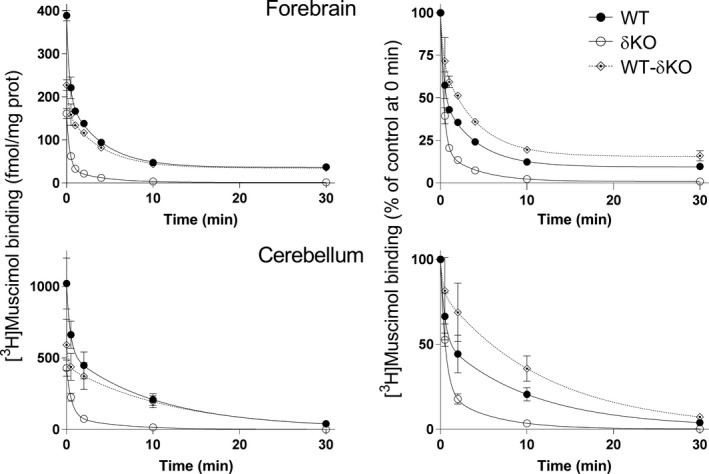
The δ subunit leads to very slow muscimol dissociation. Dissociation of 5 nM [^3^H]muscimol binding from forebrain (*n* = 4 independent experiments using individual forebrains in each experiment) and cerebellar (*n* = 3 independent experiments using pools of 3 individual cerebella from the mouse line in each pool) membranes of WT and δKO mice (mean ± SEM). The experiments were performed in triplicate technical replicates. Forebrain and cerebellar membranes of the mouse lines were pre‐incubated for 15 min with 5 nM [^3^H]muscimol alone and in the presence of 100 μM GABA to determine non‐specific binding. Then 100 μM GABA was added to all tubes to start [^3^H]muscimol dissociation. The incubations were continued for various durations (30 s to 30 min) and terminated by filtration onto GF/B filters. The values are expressed as fmol/mg protein on the left and as % of control binding at the start of dissociation (0 min) on the right. The values for δ‐GABA_A_Rs (WT‐δKO) were calculated as described in Materials and methods.

### Protein measurement

In all ligand‐binding studies, protein concentrations of membranes were determined with the Bio‐Rad Coomassie blue dye‐based protein assay kit (Hercules, CA, USA) according to manufacturer's instructions.

### Data analysis

Association and dissociation curves for estimation of association and dissociation rate constants, and saturation binding for estimation of *B*_max_ and *K*_D_ values were analyzed with Graph Pad Prism 7 software (Graph Pad, San Diego, CA, USA). Statistical significances between the groups were analyzed using unpaired *t*‐test and one‐way anova followed by Tukey's *post hoc* test Graph Pad (Graph Pad Prism 7). *p*‐values of <* *0.05 were considered significant. In this study, no sample calculation, assessment of data outliers and data normality were performed, and experiments were done unblinded.

## Results

### The majority of high‐affinity (5 nM) [^3^H]muscimol binding is because of binding to low abundance δ‐GABA_A_R

To evaluate the contribution of δ‐GABA_A_Rs to high‐affinity muscimol binding, we measured the time course of 5 nM [^3^H]muscimol binding to forebrain and cerebellar membranes from both wild‐ type and δKO mice. Deletion of the δ subunit led to > 50% reduction of 5 nM [^3^H]muscimol binding at 22°C to both forebrain and cerebellar membranes when compared to WT mice (Fig. 2). This finding is remarkable, considering that the proportion of δ‐GABA_A_Rs in the mammalian fore/midbrain is only up to 10%, depending on the exact brain region (Whiting 2003; Hörtnagl *et al*. 2013). In the cerebellum, the fraction of δ‐GABARs is close to 30% (Tretter *et al*. 2001; Pöltl *et al*. 2003), but this is accompanied by a relatively high muscimol affinity of cerebellar α6βγ2 receptors (see Fig. 1b, Mihalek *et al*. 1999; Mäkelä *et al*. 1997). The increased muscimol binding by these α6βγ2 receptors likely explains why the percent reduction in high‐affinity muscimol binding in δKO cerebellum is about the same as in the forebrain despite the much higher abundance of δ‐GABA_A_Rs in the cerebellum. Total 5 nM [^3^H] muscimol binding (fmol/mg membrane protein) was around four times higher in the cerebellum when compared to forebrain both in WT as well as in the δKO mice (Fig. 2), which is consistent with a much higher δ‐expression in the cerebellum and also a slightly higher muscimol affinity of α6βδ GABA_A_Rs (see below). Binding of 5 nM [^3^H]muscimol to non‐δ‐GABA_A_Rs in δKO forebrain was around 100 fmoles and about 300 fmoles (per mg membrane protein) in the cerebellum (see Fig. 2). Considering that generally binding to brain membranes is about ten times higher (1–2 pmol/mg membrane protein (Sieghart *et al*. 1987; Kontturi *et al*. 2011) for benzodiazepine ligands (with only one binding site, vs. two for muscimol), the amount of [^3^H]muscimol binding suggests that in the forebrain only a rather small fraction (~5%) of non‐δ‐GABA_A_Rs were occupied by muscimol under our binding conditions (Table 1).

**Table 1:** Saturation analysis of [^3^H]muscimol binding to forebrain and cerebellar membranes of WT and δKO mice at 0°C

	Apparent *B*_max_ (pmol/mg protein)	Apparent pK_D_
Forebrain membranes		
WT mice	0.66 ± 0.06	8.02 ± 0.06
δKO mice	0.41 ± 0.03**	7.68 ± 0.05**
Cerebellar membranes		
WT mice	2.2 ± 0.1	8.34 ± 0.01
δKO mice	1.8 ± 0.1*	8.15 ± 0.02**

Binding of various hot [^3^H]muscimol concentrations (0.1–30 nM) was measured in triplicate technical samples (3 for total and 3 for non‐specific binding) of WT and δKO mouse membranes at each concentration (mean ± SEM, *n* = 4 independent experiments, using individual mouse forebrains, and *n* = 4 independent experiments using samples each pooled of 3 individual cerebella from the mouse line). *p <* *0.05; **p <* *0.01, significantly different from the corresponding WT value, unpaired *t*‐test.

### High‐affinity [^3^H]muscimol binding to δ receptors is because of changes in binding kinetics, particularly very slow dissociation kinetics

To better illustrate high‐affinity muscimol binding kinetics to δ‐GABA_A_Rs, we subtracted binding from non‐δ‐GABA_A_Rs in δKO mice from binding in WT mice and also normalized the level of 5 nM [^3^H]muscimol binding to 100% at 15 min when the maximal binding was achieved (Figs 2 and 3). Both in the cerebellum and in forebrain, high‐affinity muscimol association was faster to the small fraction of high‐affinity non‐δ‐GABA_A_Rs (mostly γ2‐GABA_A_Rs) when compared to δ‐GABA_A_Rs, which was surprising since faster muscimol association would contribute to higher muscimol affinity in δ‐GABA_A_Rs. This slower muscimol association to δ‐GABA_A_Rs is reflected in higher forebrain and cerebellar association rate constants (*k*_on_) of [^3^H]muscimol binding to δKOs than to WT mouse membranes (Table 2, Fig. 2 (*p* < 0.01, unpaired *t*‐test).

**Table 2:** Association (*k*_on_) and dissociation (*k*_off_) rate constants of [^3^H]muscimol binding at room temperature in forebrain and cerebellar membranes of WT and δKO mice and in recombinant receptors expressed in HEK293 cells

	*k*_on_(M^−1^ × min^−1^)	*k*_off_(min^−1^)	*K*_D_(nM)
Forebrain membranes			
WT mice	3.3 ± 0.2 × 10^8^	0.53 ± 0.02	1.6
δKO mice	15 ± 3.1 × 10^8^ **	1.67 ± 0.15***	1.1
*WT‐*δ*KO*	1.4 ± 0.2 × 10^8^	0.23 ± 0.02	1.6
Cerebellar membranes			
WT mice	2.8 ± 0.2 × 10^8^	0.47 ± 0.14	1.7
δKO mice	7.7 ± 0.2 × 10^8^ **	1.11 ± 0.09*	1.4
*WT‐*δ*KO*	1.2 ± 0.2 × 10^8^	0.12 ± 0.03	1.0
Recombinant receptors			
α1β2	n.d.	0.49 ± 0.07###	
α1β2γ2	11 ± 0.6 × 10^8^	1.78 ± 0.18	1.6
α1β2δ	n.d.	0.18 ± 0.03###	
α6β2	n.d.	1.46 ± 0.05†††	
α6β2γ2	6.3 ± 0.5 × 10^8,^ ### ^,^ †††	0.98 ± 0.02### ^,^ †††	1.6
α6β2δ	1.0 ± 0.1 × 10^8,^ ###	0.13 ± 0.03###	1.3
α6β3δ	1.8 ± 0.1 × 10^8,^ ###	0.13 ± 0.01###	0.72

Association (*k*_on_) and dissociation rate constants (*k*_off_) of [^3^H]muscimol binding in forebrain samples (association, *n* = 8, dissociation, *n* = 4 independent experiments made using individual animal forebrains) and in samples each of pooled from 3 mouse cerebella (*n* = 3 independent experiments made using pooled samples from 3 individual animal cerebella), and in recombinant receptors (*n* = 3–6 independent experiments each performed using receptors from independent transfections/expressions) (mean ± SEM). n.d., not determined. All experiments were performed in triplicate technical replicates. Statistical comparison of forebrain and cerebellar values: *p < 0.05, **p < 0.01, ***p < 0.001, significantly different from the corresponding WT value, unpaired *t*‐test. Statistical comparison of recombinant receptor values: ###*p* < 0.001, significantly different from the corresponding α1β2γ2 value; †††*p* < 0.001, significantly different from the corresponding α6β2δ value (one‐way anova followed by Tukey's *post hoc* test).

We also looked at muscimol dissociation in WT and δKO cerebella and forebrains by evaluating high‐affinity (5 nM) [^3^H]muscimol unbinding for up to 30 min. A comparison of muscimol dissociation between WT and δKO animals shows that almost all of the slow muscimol dissociation is because of δ‐GABA_A_Rs, with only a minor component present in both the forebrain and cerebellum of δKO animals, which is because of the high‐affinity muscimol binding to non‐δ‐GABA_A_Rs (Fig. 3).

### δ‐GABA_A_Rs muscimol association (*k*_on_) and dissociation rates (*k*_off_) determine muscimol *K*_D_ values in the low nM range

After subtraction of binding to non‐δ‐GABA_A_Rs in δKO mice from binding to total GABA_A_Rs in WT mice, we were able to determine a *K*_D_ value based on the equation *K*_D_ = k_off_/k_on_. The calculated *K*_D_ value for δ‐GABA_A_Rs in the fore(mid)brain (predominantly α4βδ) is 1.6 nM, and the *K*_D_ for δ‐GABA_A_Rs in the cerebellum (α6βδ) is 1.1 nM. Therefore, under our binding conditions [5 nM [^3^H]muscimol and 22°C], the majority of δ‐receptors both in forebrain and cerebellum should be occupied at equilibrium.

We also determined dissociation rate constants of the high‐muscimol affinity component in δKOs and WT brains, although the majority of non‐δ‐GABA_A_Rs have low affinity and are therefore not occupied at 5 nM [^3^H]muscimol. Dissociation rate constants *k*_off_ of [^3^H]muscimol binding were higher in δKOs than in WTs in both forebrain (*p* < 0.001) and cerebellar membranes (*p* < 0.05) (Table 2, Fig. 3; unpaired *t*‐test) indicating faster [^3^H]muscimol dissociation in δKOs lacking δ‐GABA_A_Rs. The *K*_off_ values of the calculated *native* δ*‐GABA*_*A*_ *Rs* in both forebrain and cerebellum were smaller than those of δKO indicating slower [^3^H]muscimol dissociation from δ‐GABA_A_Rs than from γ2‐GABA_A_Rs (Table 2, Fig. 3). Both forebrain and cerebellar *k*_off_ values were also lower in calculated *native* δ*‐GABA*_*A*_ *Rs* than in WT mice.

### Association and dissociation‐binding kinetics of 5 nM [^3^H]muscimol to recombinant GABA_A_R subtypes

**Figure 4:**
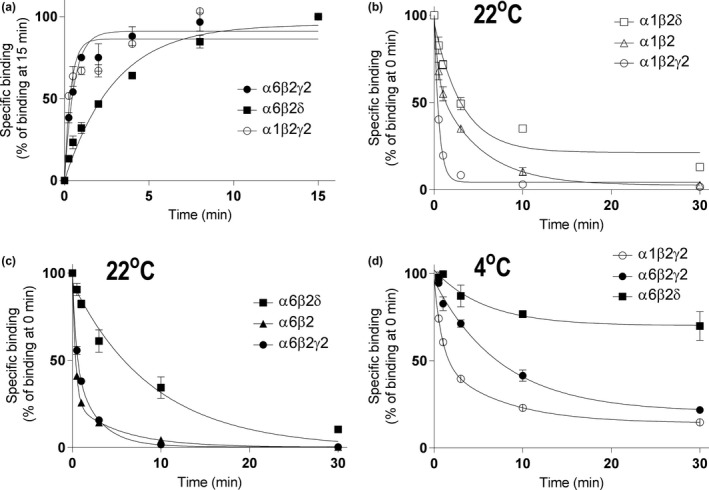
Co‐expression of the δ subunit leads to slow muscimol kinetics, particularly very slow dissociation. Association (a) and dissociation (b–d) of [^3^H]muscimol binding of recombinant α1β2γ2, α6β2γ2 and α6β2δ receptors expressed in HEK293 cells (mean ± SEM;* n* = 3–6 independent transfections and independent experiments performed in triplicate technical replicates). HEK293 cell membranes were incubated with 5 nM [^3^H]muscimol at 22°C (a–c) or on ice (d) in the absence or presence of 100 μM GABA determining the non‐specific binding. Dissociation experiments were performed as described in Materials and Methods. The incubations were terminated at various time points by filtration onto GF/B filters. The values are expressed as % of binding at 15 min (a) or 0 min (b–d).

Measurements in native brain tissues have the advantage that we can measure native receptors. The disadvantages is that the fraction of δ receptors is variable (up to 10% of α4βδ receptors in the fore/midbrain depending on brain region, up to 30% of α6βδ receptors in the cerebellum). In addition, because of the low‐muscimol affinity of most γ2‐GABA_A_R conformations, the fraction of non‐δ‐GABA_A_Rs occupied by 5 nM [^3^H]muscimol is low and probably highly variable because of differences in high‐affinity (desensitized) conformations which could also depend on subunit composition. We therefore decided to measure association and dissociation on selected recombinant receptor subtypes. As observed for *native* δ*‐GABA*_*A*_ *Rs* [^3^H]muscimol association at 22°C was much slower in α6β2δ receptors when compared to high‐affinity binding to α1β2γ2 and α6β2γ2 recombinant receptors (Table 2, Fig. 4a). The association rate constant k_on_ for α6β2δ subtype was 6.3–11‐fold lower when compared to γ2‐GABA_A_Rs (*p* < 0.001, one‐way anova).

### Dissociation of [^3^H]muscimol from recombinant GABA_A_R subtypes

Dissociation of [^3^H]muscimol from α6β2, α6β2γ2 and especially α1β2γ2 receptor subtypes was very fast (Table 2, Fig. 4b and c). Dissociation from α1β2 receptors was ‘intermediate’ while it was very slow from both the α1β2δ and α6β2δ subtypes, in αxβ2δ significantly slower than dissociation from the corresponding αxβ2γ2 subtypes (*p* < 0.01, *p* < 0.001; one‐way anova) (Table 2, Fig. 4d). From association and dissociation rates, we calculated *K*_D_ values of 0.72 nM for α6β3δ and 1.3 nM for α6β2δ GABA_A_Rs, which are in excellent agreement with the values observed with native δ‐GABA_A_Rs (see Table 2).

Since radioligand binding is frequently performed in an ice‐water bath (0°C), we decided to compare [^3^H]muscimol dissociation kinetics at 22°C with unbinding at lower temperature (0°C) on selected γ2 and δ‐GABA_A_R subtypes. At 0°C dissociation from α6β2δ and α6β2γ2 were significantly slower than from α1β2γ2 GABA_A_Rs with 70% of [^3^H]muscimol still remaining bound to α6β2δ subtype at 30 min after start of the dissociation (Table 3, Fig. 4d; *p* < 0.001). [^3^H]Muscimol dissociated also significantly slower from α6β2δ when compared to α6β2γ2 GABA_A_Rs (Table 3, Fig. 4d; *p* < 0.01, one‐way anova followed by Tukey's *post hoc* test), a difference that was also noted at 22°C (see Fig. 4c). It can be approximated that at 0°C the dissociation of [^3^H]muscimol binding during the first 15 s after pre‐incubation in recombinant α1β2γ2 receptors is about 10%, so we can assume that during the 15 s ice‐cold washing period the amount dissociated is in that magnitude for α1β2γ2 receptors and less for α6β2γ2 and α6β2δ receptors (Fig. 4d).

**Table 3:** Dissociation (*k*_off_) rate constants of [^3^H]muscimol binding at +4 °C in recombinant receptors expressed in HEK293 cells

Recombinant receptors	*k*_off_ (min^−1^)
α1β2γ2	0.352 ± 0.009
α6β2γ2	0.086 ± 0.010*** ^,^ **
α6β2δ	0.015 ± 0.003***

Dissociation rate constants (*k*_off_) of [^3^H]muscimol binding from recombinant receptors (*n* = 3 independent transfections and independent experiments performed in triplicate technical replicates. The results are expressed as mean ± SEM values). Statistical comparison of recombinant receptor values: ***p < 0.001, significantly different from the corresponding α1β2γ2 values; **p < 0.01, significantly different from the α6β2δ value (one‐way anova followed by Tukey's *post hoc* test).

The binding affinities, number of [^3^H]muscimol binding sites as well as binding kinetics are in the same range as found in the literature (Wang *et al*. 1979; Agey and Dunn 1989; Maksay 1990; Negro *et al*. 1995; Ebert *et al*. 1999). However, because of the missing high‐affinity δ‐receptors with slow kinetics, our association and dissociation rates in δKO fore/midbrain, δKO cerebellum, and recombinant α1β2γ2 receptors are an exception as they were faster than all association rates in the former published studies.

### Co‐expression of the δ subunit leads to sub‐nanomolar muscimol currents

**Figure 5:**
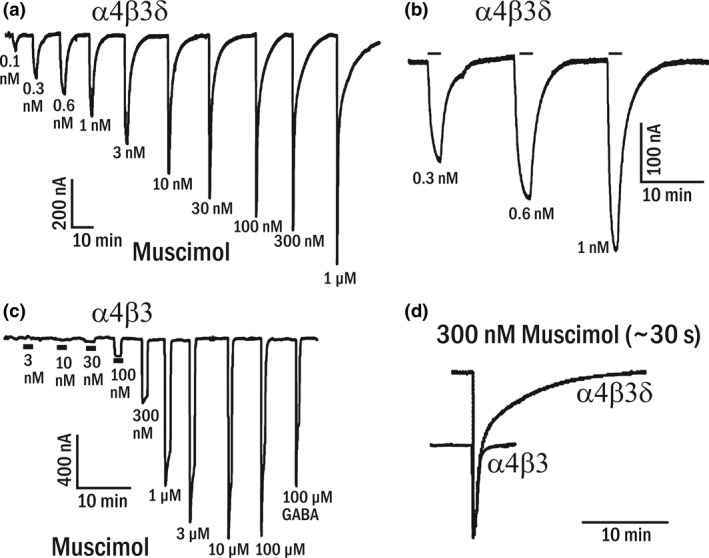
Subnanomolar concentrations of muscimol evoked currents on recombinant δ subunit‐containing GABA_A_Rs. Representative concentration‐response data (out of 3 similar recordings made using injections into different batches of oocytes) using muscimol concentrations from 0.1 nM up to 100 μM on (a) α4β3δ‐ or (c) α4β3‐injected oocytes. Muscimol concentrations from 0.1 nM to 30 nM activate currents only in α4β3δ‐injected oocytes, but not in the absence of δ subunits in α4β3 injected oocytes. (b) Slow current activation (association rates are slow at these low muscimol concentrations because association is concentration‐dependent) and also current deactivation at the lowest doses (expanded in b) and the two‐component decay for doses ≥ 10 nM. (d) Superimposed responses to 300 nM muscimol from α4β3δ‐ and α4β3‐injected oocytes. The responses were scaled so that the α4β3 300 nM muscimol current fits the fast current component in α4β3δ‐injected oocytes.

To determine the effect of muscimol on expressed recombinant receptors, we compared muscimol dose–response curves evoked with both α4β3δ and also binary α4β3 receptors. Fig. 5 shows a representative muscimol concentration‐response curve with α4 and β3 subunits either with (Fig. 5a) or without the δ subunit (Fig. 5c). Co‐expression of the δ subunit leads to receptors that respond to much lower muscimol concentrations with a threshold as low as 0.1 nM (Fig. 5a), whereas with α4β3 receptors the threshold moves to about 30 nM muscimol (Fig. 5c), indicating that δ co‐expression dramatically increases muscimol sensitivity. A closer inspection of the current traces also reveals that muscimol currents look rather different, with α4β3δ muscimol evoked currents showing a very slow return to baseline that is absent in α4β3 receptor. Such slow muscimol current deactivation is expected for a high affinity, minimally desensitizing with a slow ligand/muscimol dissociation rate as seen in our binding studies on both native and recombinant GABA_A_Rs.

In our oocyte recording chamber solution exchange takes about 1–2 s, which in many cases is not fast enough to reliably record current kinetics. However, since association rates are concentration dependent and therefore very slow at low nanomolar muscimol concentrations, they actually can be resolved under our perfusion conditions (see current close‐up in Fig. 5b), and since these are very high affinity receptors, it takes several minutes for currents to return to baseline.

## Discussion

Muscimol has long been known as a general GABA_A_R agonist, although numerous lines of evidence have emerged over the years that suggested that muscimol and also THIP, both conformationally restricted GABA analogs (see Fig. 1a) have considerable selectivity at low doses for extrasynaptic δ subunit‐containing receptors. It was shown in brain sections that δKO mice had a complete loss of 6 nM [^3^H]muscimol binding in the forebrain, with a substantial reduction in binding in the cerebellum (Mihalek *et al*. 1999, Fig. 1b). Knockout of α6 subunit (α6KO mice) lead to an essentially complete loss of high‐affinity [^3^H]muscimol binding in the cerebellum (Mäkelä *et al*. 1997, Fig. 1b). This suggested that high‐affinity muscimol binding to brain sections is δ‐ and, in the cerebellum also α6‐subunit dependent.

Binding studies are generally performed on ice (0°C), electrophysiological measurements are typically performed at 22°C and in rodent behavioral experiments receptors are studied at body temperature (37°C). Such temperature differences could have a major influence on binding affinities of GABA and GABA analogs. Also, the high‐affinity muscimol binding sites have been interpreted to represent desensitized or otherwise non‐functional high‐affinity conformations (Agey and Dunn 1989; Chandra *et al*. 2010). In addition, recombinant δ‐GABA_A_Rs so far have been shown to be fairly insensitive to muscimol requiring micromolar muscimol concentrations. Given all these uncertainties of temperature influence on binding affinity, conformational binding heterogeneity, and the absence of any evidence for highly muscimol‐sensitive functional GABA_A_Rs, it is not surprising that there is still considerable uncertainty of how muscimol affects different GABA_A_R subtypes.

We studied here GABA_A_R δKO and WT mice and recombinantly expressed GABA_A_Rs for high‐affinity 5 nM [^3^H]muscimol binding at 22°C to be able to compare them with electrophysiological data usually collected at 22°C. We show that under these conditions both in the fore/midbrain as well as in the cerebellum δKO animals lose ~60% of high‐affinity [^3^H]muscimol 22°C binding, indicating that despite their low abundance, δ‐GABA_A_Rs form the majority of high‐affinity muscimol‐binding sites in the mouse brain.

In mouse forebrain and cerebellar membranes (Fig. 3), the rate of [^3^H]muscimol dissociation was faster from δKO membranes than from WT membranes (Table 2) and both forebrain and cerebellar WT membranes have a much slower component for dissociation, that is lacking in δKO membranes. These results are corroborated by our recombinant receptor dissociation experiments, which show much slower muscimol dissociation from expressed δ‐receptors (see Fig. 4b and c). Analysis of the binding kinetics suggested that the presence of the δ subunit decreases association and even more so dissociation rates when compared to non‐δ GABA_A_R subtypes, leading to calculated dissociation constants (*K*_D_ = *k*_off_/k_on_) of 1.1 nM in the cerebellum and 1.6 nM in fore/mid‐brain (see Table 2). However, about 40% (forebrain) of high‐affinity binding remains in δKO mice with both association and dissociation faster than those observed for δ‐GABA_A_Rs (Figs 2 and 3), but in sum the calculated (from *k*_on_ and *k*_off_) apparent [^3^H]muscimol affinities (*K*_D_) for these non‐δ‐GABA_A_Rs were also around 1 nM (see Table 2). In the cerebellum, relatively high‐affinity α6βγ GABA_A_Rs likely make a major contribution to high‐affinity binding to non‐δ‐GABA_A_Rs (see Fig. 1b, Mäkelä *et al*. 1997). The fairly slow dissociation of muscimol from non‐δ‐GABA_A_Rs may help to explain differences found between [^3^H]muscimol membrane homogenate binding (Fig. 3) when compared to [^3^H]muscimol receptor autoradiography studies (Fig. 1b). During short washing procedures, only fairly small amounts of [^3^H]muscimol dissociate whereas the much longer autoradiography washing periods would allow [^3^H]muscimol to largely dissociate from non‐δ‐GABA_A_Rs (mostly α1‐5βγ2 in the forebrain) and partly also from higher affinity α6βγ2 receptors, while the extremely slow dissociation from δ‐GABA_A_Rs allows the majority of muscimol to be retained as seen in autoradiographs (Mäkelä *et al*. 1997; Korpi *et al*. 2002a,b, Fig. 1b).

The residual high‐affinity binding to non‐δ‐GABA_A_Rs in the forebrain still remains somewhat mysterious since there is no evidence for any functional muscimol responses on recombinantly expressed non‐δ‐GABA_A_Rs at low nanomolar [muscimol]. It should be noted that we estimate that < 10% of total non‐δ‐GABA_A_Rs are occupied by 5 nM [^3^H]muscimol (see Fig. 2) under our conditions in the forebrain and therefore contribute to high‐affinity binding. Since it has been reported that desensitization reversibly shifts α1β2γ2 GABA_A_Rs into a high‐affinity state (Maksay and Ticku 1984; Chang *et al*. 2002; Newell and Dunn 2002), high‐affinity muscimol binding to desensitized GABA_A_Rs (which do not contribute to muscimol‐induced currents), seems to be a plausible explanation. Another (not mutually exclusive) possibility is that such high‐affinity binding to non‐δ‐GABA_A_Rs is due to freezing, since at 22°C room temperature high‐affinity binding was lower when never‐frozen whole brain membranes were used (Yang and Olsen 1987). The notion that high‐affinity γ2‐GABA_A_R muscimol sites are non‐functional desensitized receptors and/or freezing/cooling artifacts, is consistent with the observation that behavioral low‐dose muscimol sensitivity is dependent on δ‐GABA_A_Rs (Chandra *et al*. 2010).

We show here for the first time that co‐expression of the δ subunit leads to highly muscimol‐sensitive α4β3δ currents. Remarkably, the EC_50_ for the high‐affinity muscimol component shown in Fig. 5a is in the same range as *K*_D_ for binding at 22°C. In contrast, and despite some high‐affinity binding to a fraction of non‐δ‐GABA_A_Rs, there is no evidence for highly muscimol‐sensitive currents in recombinantly expressed αβ (Fig. 5) and αβγ receptors (Adkins *et al*. 2001; Stórustovu and Ebert 2006; Mortensen *et al*. 2010). With a functional correlate missing for high‐affinity [^3^H]muscimol binding to native non‐δ‐GABA_A_Rs and recombinant γ2‐GABA_A_Rs it is possible that this high‐affinity binding to non‐δ‐GABA_A_Rs is a binding assay artifact and largely irrelevant for functional and behavioral responses. If real, that is, found in native non‐δ‐GABA_A_Rs, and not non‐functional desensitized forms, such high‐affinity binding sites could contribute, besides relatively high‐affinity α6βγ GABA_A_Rs, to behavioral high dose muscimol (and THIP) effects in δKO mice.

Recombinant expression of functional recombinant δ‐GABA_A_Rs is challenging since they generally show biphasic GABA and THIP concentration response curves likely because of incomplete δ subunit incorporation into functional receptors (Meera *et al*. 2010, 2011; Karim *et al*. 2012; Hoestgaard‐Jensen *et al*. 2014). As seen here in Fig. 5 also the muscimol concentration–response curve on α4β3δ receptors shows two components, with the low‐sensitivity component similar to what is seen with receptors formed by only α and β subunits, without δ subunits (Fig. 5) and a high affinity and slowly deactivating current component. Our highly muscimol‐sensitive δ‐GABA_A_Rs (Fig. 5) contrast with previous reports of recombinantly expressed α4/6β3”δ” receptors: Reported muscimol EC_50_ values are 200 nM on α4β3δ receptors (Mortensen *et al*. 2010), 160 nM for α6β3δ receptors and 2.28 μM on α4β3δ receptors (Stórustovu and Ebert 2006). Since these reported EC_50_ values are in the same range as we see with α4β3 receptors without δ (see Fig. 5c), they are likely the result of low δ subunit incorporation into functional receptors in recombinant expression systems. Note that our δ‐binding data, using α1β2δ and α6β2δ GABA_A_Rs shown in Fig. 4 are clear‐cut, with only little evidence of biphasic kinetic responses. A plausible and likely explanation is that with high‐affinity binding to recombinantly expressed δ‐GABA_A_Rs only a very small fraction of contaminating low‐muscimol affinity/sensitivity αβ receptors would actually be occupied at 5 nM [^3^H]muscimol.

Native and recombinantly expressed δ‐GABA_A_Rs have been suggested to be activated by relevant low ethanol concentrations (Hanchar *et al*. 2005). Given that both ethanol and muscimol are δ‐GABAR selective drugs it may not be surprising that muscimol leads to increased alcohol impairment (Frye and Breese 1982). In addition, chronic ethanol treatment leads to a substantial reduction in high‐affinity [^3^H]muscimol‐binding sites (Negro *et al*. 1995), which meshes well with the notion that chronic alcohol leads to a reduction in δ‐GABA_A_R‐mediated tonic currents and δ‐subunit surface expression, a process that likely contributes to alcohol tolerance and the development of alcohol dependence (for review see Olsen and Liang 2017).

Blood‐brain barrier (BBB) permeability usually correlates with lipid‐solubility and is therefore rather poor for highly water‐soluble molecules like GABA, muscimol and THIP. Consistent with a low BBB permeability it has been shown that only around 0.02% (1/5000) of peripherally injected [^3^H]muscimol actually entered the rat brain (Maggi and Enna 1979). High‐affinity muscimol δ‐GABA_A_Rs reported here provide a plausible explanation for brain muscimol effects, despite very low effective muscimol concentration in the brain. The program EpiSuite gives the logP (partition coefficient) value −3.60 for GABA, whereas adding hydrophobic ring structures in muscimol (logP = −1.71) and THIP (logP = −0.81) (see Fig. 1) shifts the balance from hydrophilic to more lipophilic (Estimation Programs Interface Suite^™^ for Microsoft^®^ Windows, v 4.11, United States Environmental Protection Agency, Washington, DC, USA). It seems therefore likely that GABA has the lowest BBB permeability, followed by muscimol and THIP. Given that THIP affinity for δ‐GABA_A_Rs is lower when compared to muscimol (Friemel *et al*. 2007; Meera *et al*. 2011) it is tempting to speculate that the higher BBB permeability of THIP compensates to a large extent for its much lower potency on δ‐GABA_A_Rs, with both of them having apparently very similar behavioral effects (Chandra *et al*. 2010).

The two GABA_A_ agonist binding sites in GABA_A_Rs are located at the two extracellular β+α‐ interfaces (Ernst *et al*. 2003) and so it is possible that these two GABA/muscimol binding sites do not have same affinities, and also that affinities for GABA site ligands could change once one of the sites is occupied. We show here that substitution of the γ2 by δ subunit has drastic effects on slowing [^3^H]muscimol association and even more so dissociation kinetics. While the subunit stoichiometry and organization of δ‐GABA_A_Rs has not been resolved unequivocally, there is direct evidence for a simple γ2 to δ substitution from 2α:2β:γ2 to 2α:2β:δ (Barrera *et al*. 2008). Therefore, it is likely that a 2α:2β:δ receptor would also have two GABA_A_ agonist/muscimol sites, one at each β+α‐ interface (‐β+‐α+‐δ+‐β+‐α+), without the δ subunit actually directly contributing to the GABA‐binding site. This implies that δ increases the GABA‐binding‐site affinity and slows muscimol dissociation in the βαδβα pentamer allosterically. The reciprocal of dissociation rate constant, the drug‐target residence time τ (= 1/k_off_), has been shown to often predict *in vivo* efficacy better than binding affinity (Pan *et al*. 2013; Copeland 2016) and may help explain why the δ‐subunit is required for low dose muscimol behavioral effects.

It appears that in general the GABA analog muscimol is similar to GABA in many aspects, only that it shows about 100–1000 times higher affinity (with THIP having intermediate affinity) across the board for different GABA_A_R subtypes (with α6‐containing GABA_A_Rs more sensitive). For example, muscimol EC_50_ for α1βγ2 GABA_A_Rs is ~1 μM, whereas GABA EC_50_ is ~100 μM (Karim *et al*. 2013). In contrast, for δ‐GABARs, the GABA EC_50_ is typically ~0.3–1 μM, whereas we show here that such δ‐GABARs not only bind muscimol with low nanomolar K_D_, but also that co‐expression of δ (with α4 and β3) induces low nanomolar muscimol currents.

Our results are similar to the other isoxazole GABA_A_ analog THIP, which has been shown to be highly selective for δ‐GABA_A_Rs (Meera *et al*. 2011). This paints a consistent picture in which extrasynaptic δ‐GABA_A_Rs are not only exquisitely sensitive to GABA, but also the GABA analogs THIP and muscimol. Since muscimol is a widely used experimental pharmacological tool in neuroscience research, our findings will help to better interpret *in vivo* and *in vitro* experiments that involve muscimol. While muscimol itself is unlikely to find therapeutic application, our results could help to characterize GABA analogs and GABA‐site ligands for potential therapeutic applications. For example, recent work suggested that α6βδ‐selective agonists might be useful in the clinic as antitremor medications (Handforth *et al*. 2018).

## Acknowledgments and conflict of interest disclosure

This study was supported by grants from the Finnish Foundation for Alcohol Studies (AJA, MU‐O) and NIH grant AA021213 to MW. The authors have no conflict of interest to declare.
